# HIV Infection and TLR Signalling in the Liver

**DOI:** 10.1155/2012/473925

**Published:** 2012-02-22

**Authors:** Megan Crane, Kumar Visvanathan, Sharon R. Lewin

**Affiliations:** ^1^Department of Medicine, Monash University, Melbourne, VIC, Australia; ^2^Centre for Virology, Burnet Institute, Melbourne, VIC, Australia; ^3^Department of Medicine, Monash Medical Centre, Melbourne, VIC, Australia; ^4^Infectious Disease Unit, The Alfred, Melbourne, VIC, Australia

## Abstract

Despite the availability of effective combination antiretroviral therapy (cART), liver disease is one of the leading causes of morbidity and mortality in Human Immunodeficiency Virus (HIV)-infected individuals, specifically, in the presence of viral hepatitis coinfection. HIV, a single stranded RNA virus, can bind to and activate both Toll-like receptor (TLR)7 and TLR8 in circulating blood mononuclear cells, but little is known about the effect of HIV on TLRs expressed in the liver. HIV can directly infect cells of the liver and HIV-mediated depletion of CD4+ T-cells in the gastrointestinal tract (GI tract) results in increased circulating lipopolysaccharide (LPS), both of which may impact on TLR signaling in the liver and subsequent liver disease progression. The potential direct and indirect effects of HIV on TLR signaling in the liver will be explored in this paper.

## 1. Introduction

There are 33 million people infected with human immunodeficiency virus (HIV) and despite the availability of effective combination antiretroviral therapy (cART), life expectancy in HIV-infected patients remains reduced. Liver-related mortality is now the commonest cause of non-AIDS related death in HIV-infected individuals on cART [1, 2]. Hepatitis B virus (HBV) or hepatitis C virus (HCV) are the main causes of liver disease in HIV-infected patients [3–6] with more rapid liver disease progression and higher liver-related mortality compared with individuals infected with either HBV or HCV alone [3–6].

Recently, there have been increasing reports of liver disease in HIV-infected individuals in the absence of viral hepatitis [7–11]. These diseases largely include liver decompensation with and without evidence of cirrhosis, nonalcoholic liver disease (NALD) its more severe form nonalcoholic steatohepatitis (NASH) and hepatocellular cancer (HCC) [7–11]. Liver disease in HIV monoinfection has been significantly associated with high HIV RNA, prolonged exposure to cART as well as high body mass index (BMI), alcohol abuse and increasing age [12–14].

This paper will discuss the likely direct and indirect effects of HIV on the innate immune system of the liver and the potential contribution of these changes to liver disease in the presence and absence of coinfection with hepatitis B and C.

## 2. Toll-Like Receptor (TLR) Signalling and the Liver

Toll-like receptors (TLRs) are pattern recognition receptors that recognize pathogen-associated molecular patterns (PAMPS). All TLRs have a cytosolic Toll/IL-1 receptor (TIR) domain, which is responsible for signal transduction. Myeloid differentiation primary response gene 88 (MyD88) is the main TIR-domain containing adapter molecules common to all TLRs except TLR3. Other TIR-domain containing adapter molecules include Toll/IL-1 receptor domain containing adaptor protein (TIRAP), Toll/IL-1 receptor domain containing adaptor inducing interferon-beta (TRIF), and TRIF-related adaptor molecule (TRAM) [15–17] (Table 1). Triggering TLRs leads to activation of nuclear factor kappa B (NF*κ*B) resulting in an upregulation of proinflammatory cytokines such as Tumour Necrosis Factor (TNF)-*α*, IL-1 and IL-6, and/or activation of Interferon Response Factors (IRFs) which mediate transcription of Interferon (IFN) *α* and *β* and subsequent downstream IFN-stimulated genes (ISGs). The specificity of the TLR signalling response is critically regulated by the various TIR-domain containing adapter molecules that associate with the TLR directly or colocalise with MyD88 [15–17].

MessengerRNA (mRNA) for all ten TLRs are expressed in murine livers, and expression levels can be modulated with corticosteroid treatment [18]. All ten human TLRs (TLR 1-10) are expressed in the liver with differential expression depending on the cell type [17]. While the liver is constantly exposed to bacterial microflora resident in the gastrointestinal (GI) tract via the portal circulation the inflammatory response is generally averted in the healthy liver by modulation of the innate immune response, referred to as “immune tolerance” (as reviewed by [19–21]). However, under conditions of chronic bacterial exposure, TLR expression may be similarly altered [22].

Primary cultured hepatocytes express TLRs 1–10 although the expression levels of TLRs 2–5 are very low and may indicate immune tolerance or modulation of TLR expression in response to bacterial exposure [21].

Kupffer cells express TLRs 2, 3, 4, and 9 and are the first cell type in the liver to recognise translocated bacterial products, such as lipopolysaccharide (LPS) from the GI tract [21]. The physiological response of Kupffer cells to LPS in a normal healthy person is also hyporesponsive or “tolerant” [21]. Stimulation of TLR4 triggers a proinflammatory, profibrotic response with production of chemokines CCL2, 3 and 4, adhesion molecules such as vascular cell adhesion molecule 1(V-CAM 1) and intercellular adhesion molecule 1(I-CAM 1), transforming growth factor-*β* (TGF*β*) and upregulation of TLR2 expression [23–25].

Hepatic stellate cells (HSC) also express TLRs 4 and 9 [23, 24]. The response of HSC to TLR9 ligation is profibrotic with enhanced collagen expression, but ligation may also limit fibrosis by inhibiting HSC migration [24].

Other main resident cell types in the liver also express TLRs including TLR2–5 on human biliary epithelial cells [26] and TLR4 on rat liver sinusoidal endothelial cells (LSEC) [27]. Biliary epithelial cells and LSEC are generally tolerant to LPS stimulation of TLR4 [26, 27]. Liver dendritic cells (DC) and natural killer cells (NK) express a wide range of TLRs including TLR2, 3, 4, 7, and 9 on DCs and TLR1, 2, 3, 4, 6, 7, and 9 on NK cells [17]. Unlike the resident monocytic kupffer cells, DCs and NKs respond appropriately to TLR4 ligation under normal physiological conditions [17].

## 3. TLR Signalling, the Liver and HIV

### 3.1. Direct Effects

#### 3.1.1. HIV Infection of Liver Cells

Multiple cells in the liver can be infected with HIV. HIV RNA has been detected in primary human hepatocytes both *ex vivo* [28–30] and *in vitro* [31, 32], and we have recently shown that a number of hepatocyte cell lines are permissive to low level HIV infection *in vitro *[33]. Kupffer cells can be infected by HIV *in vivo* [28–30] and *in vitro* studies suggest that HIV infection of primary Kupffer cells leads to productive infection [31, 32]. Binding of HIV or glycoprotein (gp)120 to CXCR4 expressed on HSCs led to an increase in CCL2 and other markers of HSC activation including alpha smooth muscle actin (*α*SMA) and TGF-*β* [34, 35].

#### 3.1.2. Ligation of TLR7 and 8 by HIV

Given HIV RNA is also a TLR7/8 ligand [36] and hepatocytes express either TLR7, TLR8, or both, HIV may also directly activate TLRs in the liver, although to date this has not been explored.

**Figure 1:**
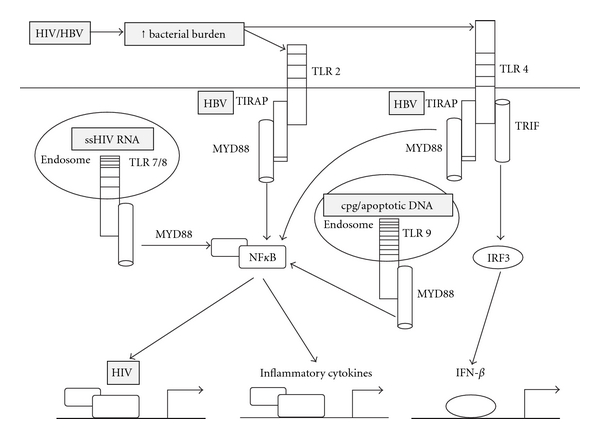
TLR signalling in the liver in the setting of HIV-HBV coinfection. HIV infection results in a significant increase in circulating LPS, potentially triggering an inflammatory response via activation of TLR4. ssHIV RNA can also activate TLR7 or 8 leading to an increase in NF-*κ*B which can drive both HIV replication and production of proinflammatory cytokines and chemokines. HBV binds to and inhibits signalling through Mal/TIRAP which would inhibit proinflammatory responses in the coinfected liver. Increased LPS and/or increased hepatic apoptosis in the coinfected liver could also activate TLR9 resulting in further inflammation and HIV replication.

The effects of HIV on TLR activation have been assessed in mononuclear cells from blood. Ligation of TLR8 by HIV RNA led to an increase in HIV replication and TNF-*α* production in monocytes from blood via activation of the MyD88-dependent NF*κ*B pathway [37, 38]. Following HIV infection of monocytes or stimulation with single stranded (ss)HIV RNA the response to LPS stimulation was significantly enhanced with an increased production of proinflammatory cytokines including TNF-*α*, consistent with a loss of “tolerance” [39]. Given Kupffer cells are of monocytic origin, they may also respond to HIV through activation of TLR7/8 (Figure 1). Therefore, if there was any low level persistence of HIV RNA in the liver, even in the setting of suppressive cART, this may potentially enhance the intrahepatic inflammatory responses to LPS.

#### 3.1.3. TLR9 and HIV

Other TLRs, such as TLR9, may also contribute to effects of HIV on the liver. Several studies have now shown TLR9 polymorphisms are associated with rapid disease progression [40, 41] and higher peak viral load [42] suggesting TLR9 ligation events are important drivers of inflammation during HIV infection.

TLR9 binds CpG-DNA of bacterial origin but more recently, it has been shown that TLR9 can also be activated by hepatocyte-derived apoptotic DNA fragments [24]. HIV can induce hepatocyte apoptosis *in vitro* via binding of gp120 to CXCR4 even in the absence of productive infection [43]. We have recently shown that intrahepatic apoptosis is increased in the setting of HIV-HBV coinfection using immunohistochemistry of liver biopsies [44]. Signalling through TLR9 has also been shown to suppress HIV replication in *ex vivo* lymphoid tissue blocks which correlated with production of chemokines such as CXCL-10 and 12 and CCL 3, 4, and 5 [45]. Therefore, indirect effects of HIV on TLR9 may also contribute to liver disease in the setting of HIV infection.

#### 3.1.4. HIV and Dendritic Cells

Dendritic cells provide an important bridge between the innate and adaptive immune systems. Plasmacytoid DC (pDC) in particular secrete type I IFNs and proinflammatory cytokines following stimulation with either TLR7 or TLR9 that in turn activate T cells. During HIV infection, DC numbers and function are altered [46]. HIV gp120 has been shown to inhibit the pDC proinflammatory response to TLR9 ligation *in vitro* [47], and it has been demonstrated that ssHIV RNA activates pDCs via TLR7/8 ligation suggesting a mechanism whereby HIV drives chronic immune activation [36]. However, the inflammatory response of DCs from HIV-infected patients to TLR ligation is unclear and has been described as either reduced [48, 49] or unchanged [50, 51]. It is not currently known how HIV or TLR ligation affects DCs of the liver.

### 3.2. Indirect Effects: HIV and LPS

HIV infection leads to a significant depletion of CD4 T cells in the GI tract which is associated with a significant increase in plasma LPS [52–54]. It is hypothesised that the increased LPS burden in the setting of HIV infection leads to activation of the innate immune response including monocyte/macrophages and DCs leading to a significant increase in IFN-*α*, IL-6, and TNF-*α* [55]. There is also a significant association between LPS and circulating activated CD4 and CD8 T cells (as measured by expression of CD38 and HLA-DR) [53]. Exposure of peripheral blood mononuclear cells (PBMCs) to agonists of TLRs 3, 4, 5, and 9 *in vitro* resulted in activation of both memory and effecter CD4 and CD8 T cells, consistent with LPS also driving T-cell activation [56].

While it is currently unclear if elevated LPS plays a role in liver disease progression in HIV monoinfection, it is clear that elevated LPS is associated with, and in some settings contributes to, liver disease progression, for example, alcoholic liver disease [57], nonalcoholic fatty liver disease, nonalcoholic steatohepatitis [58–60], chronic hepatitis C virus (HCV) infection [61, 62], chronic HBV [61], and HIV-HCV coinfection [63]. Increased intestinal permeability in HIV-HCV coinfected patients with cirrhosis was associated with an increase in circulating LPS and monocyte activation in blood and an increase in gene expression of hepatic TLR2 and TLR4 [22]. In addition, TNF-*α* expression was significantly correlated with hepatic inflammation as measured by histology [64].

NF*κ*B is a common downstream component of the TLR signalling pathway. The HIV promoter, referred to as the long terminal repeat (LTR), contains two NF*κ*B binding sites, and HIV transcription is significantly increased following NF*κ*B binding [65]. This can be negatively regulated by nuclear receptors peroxisome proliferator-activated receptor gamma (PPAR*γ*) and liver X receptor (LXR) [66]. Several studies have shown that TLR activation by microbial products such as LPS or flagellin enhances HIV replication via an increase in NF*κ*B [45, 67], therefore, a positive feedback mechanism may exist whereby elevated circulating LPS drives HIV replication which may drive further CD4 T-cell depletion in turn driving further increases in LPS (Figure 1).

Strategies that target immune activation in HIV infection might also be potentially beneficial in the management of liver disease the setting of HIV infection. Amongst many antiactivation approaches that are currently being evaluated, hydroxychloroquine, a drug used to treat inflammatory arthritis, shows the most promise. Hydroxychloroquine reduces endosomal TLR signalling and has recently been evaluated in HIV-infected patients with reduced CD4 T-cell recovery [68]. In this study, hydroxychloroquine led to a significant decrease in plasma LPS and multiple other markers of immune activation and also led to enhanced CD4 T-cell recovery [68]. Further work is still required to better understand whether reducing immune activation in HIV-infected patients on cART will have an effect on liver disease outcomes.

## 4. TLR Signalling in HIV-HBV Coinfection: Potential Interactions

On average, 10% of patients infected with HIV are coinfected with hepatitis B virus. With the introduction of cART, which also includes agents active against both HIV and HBV (HBV-active HAART), liver-related mortality rates have reduced [1] but total and liver-related mortality still remain significantly elevated in HIV-HBV coinfected patients [6, 69].

In a mouse model of HBV infection, activation of TLRs 3, 4, 5, 7, or 9 suppressed HBV replication [70] and in another study activation of TLR3 and TLR4 suppressed HBV replication via the MyD88-independent pathway [71]. Like most successful persistent viruses, HBV has evolved to adapt and even subvert the host innate immune response.

### 4.1. HBV and TLR2 and TLR4

Previous work by ourselves and others has shown that in chronic HBV monoinfection, TLR2 expression was downregulated on primary circulating monocytes in chronic HBV infection compared to uninfected controls [72–74]. Compared to HBeAg-negative patients, HBeAg positive patients had lower expression of TLR2 on monocytes, hepatocytes and kupffer cells [74]. This was associated with a decrease in TNF-*α* expression when either TLR4 or TLR2 was activated *in vitro* [74]. Most recently, in an hepatocyte cell line, we have shown that HBeAg binds to TIR-domain containing adapter-inducing interferon (TRIF)-related adapter molecule (TRAM) and MyD88-adapter like/Toll/IL-1 receptor domain containing adapter protein (Mal/TIRAP) to effectively inhibit signalling through TLR2 and TLR4 which may represent another strategy for HBV to evade the innate immune response [75].

In HIV-HBV coinfection, it is plausible that chronic exposure to elevated LPS could contribute to a loss of tolerance in TLR4 signalling in the liver. In combination with an impaired TLR2 signalling response mediated by HBV, the TRIF-dependent IFN response might be further enhanced (Figure 1). These interactions warrant further investigation using *in vitro* model systems.

### 4.2. HBV and TLR9

HBV has also been shown to impair TLR9 signalling in plasmacytoid DCs (pDCs) leading to reduced IFN-*α* production [76] which may be another mechanism for evasion of the immune system. TLR9 expression is increased in patients with chronic HBV compared to uninfected controls, and TLR9 expression significantly correlates with HBV DNA levels in plasma [77], suggesting a link between TLR9 expression and viral replication. Given the direct and indirect effects of HIV on TLR9, it is possible that, in the setting of HIV-HBV coinfection, TLR9 signalling in the liver by both HIV and HBV could result in an increase in chemokine production and a proinflammatory response.

## 5. TLR Signalling in HIV-HCV Coinfection: Potential Interactions

The role of TLR signalling in HCV infection has been reviewed elsewhere [23]. In brief, HCV has adapted multiple methods of evading the host innate immune response by downregulating signalling through TLRs 2, 4, 7, and 9 by binding to and interfering with MyD88 and TRIF in multiple cell types including hepatocytes, macrophages, and plasmacytoid (p)DCs [23]. HCV has also been shown to cleave mitochondrial antiviral signalling protein (MAVS) which forms part of the viral sensing TLR signalling cascade, inhibiting IFN-*α*/*β* production, and transcription of antiviral ISGs [78]. Recent studies have also shown a potential role for TLR7 and 9 agonists in promoting clearance of HCV [79–81]. This would suggest that HCV would contribute to a reduction in the antiviral response in patients with HIV-HCV coinfection.

We recently examined liver RNA and peripheral blood monocytes from patients with HCV monoinfection (*n* = 46), HIV-HCV coinfection (*n* = 27), and HIV monoinfection (*n* = 20). We found that increasing Metavir inflammatory activity score was associated with increased hepatic TLR2 and TLR4 mRNA as measured by reverse transcriptase quantitative PCR (RT-qPCR) [64]. We also found a significant correlation between hepatic mRNA expression of TNF-*α* and TLR2 and TLR4. Interestingly, we found no differences in TLR mRNA or protein expression between HCV monoinfected, HIV-HCV coinfected, or HIV monoinfected patients [64].

Monocytes from HCV and HIV-HCV coinfected patients lack LPS tolerance and this lack of tolerance correlated with liver inflammation, as measured by elevated intrahepatic Kupffer cells activation markers CD163 and CD33 and elevated circulating aspartate aminotransferase [61–63]. One study suggested an impaired or tolerant response to TLR3 and TLR4 in monocytes from HIV-HCV-infected individuals when stimulated *ex vivo*, however, in this study high spontaneous secretion of IL-6 and TNF-*α* were observed, suggesting a loss of tolerance *in vivo* [82]. Plasma levels of LPS in HIV-HCV-coinfected patients also correlated with severity of cirrhosis [63], where patients with LPS in the upper quartile (>42 pg/mL) had a 19-fold higher risk of disease progression compared to patients with LPS in the lower quartile.

## 6. Conclusion

HIV infection of the liver could potentially enhance TLR signaling via a direct effect of either the virus itself or HIV-related proteins as well as via an indirect effect on increased circulating LPS levels. Our understanding of how HIV affects TLR function is largely derived from studies in blood. Further studies are needed to define the effects of HIV on expression of TLR and functional TLR signaling in hepatic cells, specifically in the presence of HBV and HCV coinfection. These studies will be critical for the development of novel strategies to manage HIV-HBV and HIV-HCV coinfection that extend beyond the use of antivirals alone.

## Figures and Tables

**Table 1:** Known TLRs, ligands for these TLRs and the relevant signalling pathways.

TLR	Recognises	Signals through
1	Triacyl lipopeptides	TIRAP-MyD88
2	Tri- and diacyl lipopeptides	Mal/TIRAP-MyD88
3	dsRNA	TRIF-MyD88 independent
4	LPS	Mal/TIRAP-MyD88 & TRIF- MyD88 independent
5	Flagellin	MyD88
6	Diacyl lipopeptides	TIRAP-MyD88
7	ssRNA	MyD88
8	ssRNA	MyD88
9	CpG-DNA (bacterial origin and host derived apoptotic DNA fragments)	MyD88
10	Undefined	Undefined
